# Baseline cardiovascular comorbidities, and the influence on cancer treatment decision-making in women with breast cancer

**DOI:** 10.3332/ecancer.2021.1293

**Published:** 2021-09-21

**Authors:** Shridevi Subramaniam, Yek-Ching Kong, Hafizah Zaharah, Cuno S.P.M. Uiterwaal, Andrea Richard, Nur Aishah Taib, Azura Deniel, Kok-Han Chee, Ros Suzanna Bustamam, Mee-Hoong See, Alan Fong, Cheng-Har Yip, Nirmala Bhoo-Pathy

**Affiliations:** 1 Department of Social and Preventive Medicine, Faculty of Medicine, Universiti Malaya, 50603 Lembah Pantai, Kuala Lumpur, Malaysia; 2 Centre of Clinical Epidemiology, Institute for Clinical Research, National Institutes of Health, Setia Alam, 40170 Shah Alam, Malaysia; 3 Centre for Epidemiology and Evidence-Based Practice, Faculty of Medicine, Universiti Malaya, 50603 Lembah Pantai, Kuala Lumpur, Malaysia; 4 Department of Radiotherapy & Oncology, National Cancer Institute, 62250 Putrajaya, Malaysia; 5 Julius Center for Health Sciences and Primary Care, University Medical Center Utrecht, 3508 GA Utrecht, The Netherlands; 6 Department of Surgery, Faculty of Medicine, Universiti Malaya, 50603 Lembah Pantai, Kuala Lumpur, Malaysia; 7 Department of Radiotherapy and Oncology, Hospital Kuala Lumpur, 50586 Kuala Lumpur, Malaysia; 8 Cardiology Unit, Department of Medicine, Faculty of Medicine, Universiti Malaya, 50603 Lembah Pantai, Kuala Lumpur, Malaysia; 9 Sarawak Heart Centre, 94300 Kota Samarahan, Sarawak, Malaysia; 10 Subang Jaya Medical Centre, 47500 Subang Jaya, Selangor, Malaysia

**Keywords:** breast cancer, cardiovascular risk factors, cardiotoxicity

## Abstract

**Purpose:**

To measure the baseline prevalence of cardiovascular disease (CVD), its modifiable and non-modifiable risk factors in breast cancer patients, and determine their association with adjuvant treatment decision-making.

**Method:**

From 2016 to 2017, 2,127 women newly-diagnosed with breast cancer were prospectively recruited. Participants’ cardiovascular biomarkers were measured prior to adjuvant treatment decision-making. Clinical data and medical histories were obtained from hospital records. Adjuvant treatment decisions were collated 6–8 months after recruitment. A priori risk of cardiotoxicity was predicted using the Cardiotoxicity Risk Score.

**Results:**

Mean age was 54 years. Eighty-five patients had pre-existing cardiac diseases and 30 had prior stroke. Baseline prevalence of hypertension was 47.8%. Close to 20% had diabetes mellitus, or were obese. Dyslipidaemia was present in 65.3%. The proportion of women presenting with ≥2 modifiable CVD risk factors at initial cancer diagnosis was substantial, irrespective of age. Significant ethnic variations were observed. Multivariable analyses showed that pre-existing CVD was consistently associated with lower administration of adjuvant breast cancer therapies (odds ratio for chemotherapy: 0.32, 95% confidence interval: 0.17–0.58). However, presence of multiple risk factors of CVD did not appear to influence adjuvant treatment decision-making. In this study, 63.6% of patients were predicted to have high risks of developing cardiotoxicities attributed to a high baseline burden of CVD risk factors and anthracycline administration.

**Conclusion:**

While recent guidelines recommend routine assessment of cardiovascular comorbidities in cancer patients prior to initiation of anticancer therapies, this study highlights the prevailing gap in knowledge on how such data may be used to optimise cancer treatment decision-making.

## Introduction

Cardiovascular disease (CVD) is emerging to rival cancer recurrence as a leading cause of death in women with early breast cancer [1]. The increased risk of morbidity and mortality from CVD in women with breast cancer may be attributed to a combination of the direct cardiotoxic effects (e.g. anthracycline-based chemotherapy, trastuzumab, radiotherapy) and the indirect effects (e.g. weight gain, loss of cardiorespiratory fitness) of cancer therapy, coupled with clustering of conventional risk factors of CVD including hypertension, diabetes mellitus, dyslipidaemia, obesity and smoking [2–8].

Until very recently, guidelines for cardiovascular risk assessment in adults with cancer had been scarce [9–10]. Particularly, routine screening for conventional risk factors of CVD at initial cancer diagnosis had been lacking in oncology practices worldwide [9] despite its potential to facilitate cardiovascular profiling, adjuvant treatment decision-making and identification of high-risk patients requiring early cardiology referral and close monitoring [11]. In recognition of the increasing need for guidance, the American Society of Clinical Oncology had in 2017 put up a specific guideline for cardiovascular care of cancer patients, which was endorsed by the American College of Cardiology and the American Heart Association. This guideline recommends physicians to perform comprehensive cardiovascular evaluation in adult patients with cancer including screening for modifiable risk factors of CVD before initiation of potentially cardiotoxic therapies [12–13].

Through an inception cohort study, we measured the prevalence of CVD, its modifiable (hypertension, diabetes, obesity, dyslipidaemia, smoking) and non-modifiable (family history) risk factors, prior to administration of anticancer therapies in women newly diagnosed with breast cancer in a multiethnic setting. Importantly, amidst the lack of evidence-based standards, we assessed whether the baseline presence of CVD or presence of multiple risk factors of CVD was associated with adjuvant treatment administration, to gain an insight on whether these factors currently influenced cancer treatment decision-making in routine clinical practice. *A priori* risk of cardiotoxicity following adjuvant therapy was also predicted.

## Patients and methods

### Study population

The study population comprised women who were newly diagnosed with stage I up to stage IV breast cancer between January 2016 and December 2017. Four different hospitals were selected across Klang Valley, an urban conglomeration in Malaysia, to allow recruitment of a demographically diverse sample of patients; National Cancer Institute (national oncology referral center), Kuala Lumpur Hospital (public general hospital), University Malaya Medical Centre (public university hospital) and Subang Jaya Medical Centre (private medical centre). In total, 2,127 females with a first time histological-diagnosis of breast cancer were consecutively recruited within 6-8 weeks of diagnosis, prior to initiation of cancer therapy. Patients with recurrent breast cancer were excluded.

### Ethical approval

Ethics approval was obtained from the relevant institutional committees. Written informed consent was obtained from all participants. Ethical principles according to Helsinki’s declaration were observed throughout the conduct of the study.

### Data collection and study variables

Participants were recruited during their hospital visits. Data on ethnicity (Malay, Chinese, Indian or other race), age at diagnosis, highest-attained education (primary, secondary or tertiary) and medical history (prior diagnosis with hypertension, diabetes mellitus, dyslipidaemia, eCVDs (myocardial infarction, angina pectoris, unstable angina, heart failure and stroke), other comorbidities and medications) were collected via face-to-face interviews. Data on smoking status and family history of premature heart disease (history of coronary heart disease before the age of 60 years in first-degree relatives) were also gathered. All medical and drug histories were verified with medical records.

During the on-site screenings, height and body weight were measured. Body mass index (BMI) was calculated as weight divided by height squared (kg/m^2^). Obesity was recorded if the calculated BMI was 30 kg/m^2^ and above. Systolic and diastolic blood pressure readings were taken using automated sphygmomanometers. Hypertension was recorded based on self-report or if patients were on hypertensive medication and/or had a repeatedly elevated BP reading; systolic blood pressure >140 mmHg or diastolic blood pressure >90 mmHg (measured at least twice).

Non-fasting blood samples were drawn to measure lipid and glucose levels. Dyslipidaemia was recorded based on self-report or if patients were on cholesterol lowering medication and/or had a serum total cholesterol level ≥ 5.2 mmol/L and/or serum high density lipoprotein levels (HDL) < 1.3 mmol/L [14]. Diabetes status was recorded based on self-report or if patients were on diabetes medication and/or had a non-fasting blood glucose concentration of ≥11.0 mmol/L.

Patients who were newly detected with abnormalities in their blood pressure or serum glucose/lipid levels were provided with referral letters for further management.

Data on tumour characteristics were obtained from hospital records (date of diagnosis, histology, tumour size at presentation, number of positive axillary lymph nodes, tumour grade (grade I, grade II, grade III), distant metastasis at initial diagnosis (yes or no), TNM stage (I, II, III, IV), progesterone and oestrogen receptor status (positive, negative) and human epidermal growth factor receptor 2 status (positive, negative)).

### Follow-up for adjuvant treatment administration, and prediction of cardiotoxicity

Patients’ baseline data on risk factors of CVD were accessible through the medical records to all treating physicians prior to adjuvant treatment decision-making. Data on administration of type of surgery, radiotherapy, chemotherapy, chemotherapy regimen, endocrine therapy, type of endocrine therapy, targeted therapy and type of targeted therapy were retrieved from the hospital records within 6–8 months after recruitment.

Individual risk of cardiotoxicity was subsequently calculated using the Cardiotoxicity Risk Score, a risk-stratification tool introduced by the Mayo Clinic [11] that allows physicians to assign cancer patients into very low, low, intermediate, high and very high cardiotoxicity risk groups based on a set of patient-and cancer treatment-related risk factors. Under this scoring system, one point was assigned for a medical history of hypertension, diabetes, cardiomyopathy or heart failure, having a history of coronary artery disease, age below 15 or above 65 years, being female, prior or concurrent anthracycline-based chemotherapy and prior or concurrent chest radiation. Up to four points were assigned if patients received cardiotoxic drugs such as cyclophosphamide, anthracycline or trastuzumab. A total score of less than two was considered to indicate a low risk of cardiotoxicity, while a score of three to four represented an intermediate risk, a score of five to six was deemed high risk and a score of more than six denoted a very high risk [11].

### Data analysis

Categorical variables were summarised using frequency and percentages. Continuous variables were expressed in mean ± standard deviation. Overall prevalence of cardiovascular comorbidities with 95% confidence intervals (CIs) was estimated for participants, followed by stratification by age and ethnicity.

Among patients with non-metastatic disease, multivariable logistic regression analyses were used to determine the association between preexisting CVD, family history of premature heart disease and clustering of risk factors of CVD (presence of ≥2 modifiable risk factors) with administration of individual modalities of adjuvant treatment. Models were adjusted for age (years), ethnicity (Chinese, Malay, Indian, others), type of hospital (public, private, university), tumour size (pT1, pT2, pT3, pT4), lymph node status (pN0, pN1, pN2, pN3), oestrogen/progesterone receptor status (positive, negative), human epidermal growth factor receptor 2 status (positive, negative), tumour grade (I, II, III), adjuvant chemotherapy administration (no, yes), adjuvant radiotherapy administration (no, yes), adjuvant endocrine therapy administration (no, yes) and trastuzumab administration (no, yes).

Complete case analyses were performed, and multiple imputation was employed as a form of sensitivity analysis.

## Results

In this large cohort of multiethnic Asian women (Chinese: 47.9%, Malays: 37.4%, Indians: 12.7%) with breast cancer, the mean age at diagnosis was 54 years (11.6). Mean tumour size at presentation was 3.6 cm (2.9). Overall, 22.4% presented with stage I disease, while 38.9%, 27.9% and 10.8% presented with stage II, stage III and stage IV breast cancers, respectively. A vast majority (70%) had oestrogen receptor positive tumours. Among 1,876 patients with non-metastatic breast cancer, 63.1% received chemotherapy (53.0%; anthracycline- and taxane-based regimens, 41.4%; anthracyclines only, 5.2%; taxanes alone), 63.1% received adjuvant radiotherapy, while approximately 20% of patients (126/618) with human epidermal growth factor receptor 2 (HER2) positive tumours received trastuzumab (not shown). Missing values ranged between 0.2% (diabetes mellitus) and 12.1% (tumour grade).

Eighty-five patients had preexisting cardiac diseases (4.0%, 95% CI: 3.2%–4.8%). Of these, a majority comprised coronary heart disease (*n* = 75), with 31 patients having suffered a previous myocardial infarction (Table 1). Thirty patients had a prior history of stroke. Approximately 11% of patients reported a family history of premature coronary heart disease. Very few patients were classified as having a cardiovascular risk factor based on self-report alone (hypertension: 15 patients, dyslipidaemia: 17). The baseline prevalence of hypertension in this cohort of women with newly diagnosed breast cancer was 47.8% (95% CI: 45.7%–49.9%) (Table 1). Close to 20% of patients had diabetes mellitus at initial breast cancer diagnoses (18.8%, 95% CI: 17.1%–20.5%). Prevalence of obesity at baseline was 18.0% (16.3%–19.6%). A majority (65.3%) of patients presented with dyslipidaemia; 44.7% (95% CI: 42.3%–47.1%) had elevated total cholesterol, 38.1% (95% CI: 35.7%–40.4%) had low HDL levels (not mutually exclusive) (not shown). Only a minority reported smoking (1.7%, 95% CI: 1.1%–2.2%). In this relatively young cohort of multiethnic women with breast cancer, the clustering of risk factors of CVD was substantial where 26.3% of patients presented with two modifiable risk factors at baseline, while another 20.7% already had three risk factors at initial breast cancer diagnosis. Strikingly, the clustering of CVD risk factors was not negligible even among the very young breast cancer patients. All the cardiovascular profiles were significantly associated with age (*p* value < 0.05) except smoking and family history of early-onset coronary heart disease (Table 1). Significant ethnic variations were observed, whereby Indian women with breast cancer were more likely to have preexisting CVD, diabetes, hypertension, dyslipidaemia and family history of early-onset coronary heart disease compared to their Chinese and Malay counterparts (*p* value < 0.05) (Table 2). Clustering of modifiable risk factors for CVD remained highest in Indian patients with breast cancer, with 63.6% presenting with two or more risk factors, compared to the Malays (54.4%) and the Chinese (36.5%).

In the multivariable analyses examining the association between cardiovascular profile and adjuvant treatment administration, presence of pre-existing CVD was significantly associated with lower odds of receiving chemotherapy (adjusted odds ratio (OR): 0.32, 95% CI: 0.17–0.58). None of the patients with pre-existing CVD received trastuzumab. Radiotherapy or endocrine therapy administrations were also less likely in women with pre-existing CVD, albeit not reaching statistical significance (Table 3). Interestingly, the presence of multiple modifiable risk factors of CVD at initial diagnosis was not associated with administration of most adjuvant therapies except endocrine therapy (Table 3).

Using the Cardiotoxicity Risk Score, approximately one fifth of breast cancer patients were predicted to have low risk of cardiotoxicity (19.9%), while a majority appeared to have high (33.7%) or very high risks (29.9%) of developing cardiotoxicities (Table 4). Patients aged <40 years, those of Malay and Indian ethnicities, as well as women with more advanced cancer stages, appeared to be more vulnerable. Multivariable analysis further revealed that younger age and advanced cancer stages remained independently associated with a higher predicted risk of cardiotoxicity.

Results from sensitivity analyses using multiple imputation did not change the study inferences.

## Discussion

Findings from this multi-ethnic setting revealed a high baseline burden of modifiable risk factors of CVD among women presenting with breast cancer, including in those who were very young. While pre-existing CVD appeared to be consistently associated with lower administration of adjuvant breast cancer therapies, presence of multiple risk factors of CVD at initial cancer diagnosis did not seem to influence adjuvant treatment decision-making. This, however, must be considered in light of our findings that a high burden of cardiovascular comorbidities at baseline coupled with administration of anthracyclines puts women with breast cancer at high *a priori* risks of cardiotoxicity.

The baseline prevalence of hypertension, diabetes and dyslipidaemia among the multiethnic breast cancer patients in this study appears to be substantially higher than previously reported [10, 15–17]. This may be explained by the high burden of risk factors of CVD in the background multiethnic population of Southeast Asia as have been reported in Malaysia, as well as Singapore [18–19]. The present observations warrant concern given that in the Life after Cancer Epidemiology Study, hypertension was independently associated with an increased risk of all-cause mortality among early-stage breast cancer survivors due to causes both related and unrelated to cancer [20]. A previous study had also shown that breast cancer patients with diabetes were more likely to be hospitalised for chemotherapy-induced toxicity than their counterparts without diabetes, leading to higher cancer specific mortality (hazard ratio (HR):1.20, 95% CI: 1.07–1.35) [21]. More recently, Hershman *et al* [22] through an analysis of data from clinical trials of women with breast cancer had shown that presence of every additional risk factor of CVD at baseline was associated with increased incidences of cardiac events and mortality.

The ethnic differences in prevalence of risk factors of CVD, and predicted risks of cardiotoxicity among women with breast cancer in the present study to some extent may explain the disparities in survival following breast cancer between the Malay, Indian and Chinese patients that we had previously reported from Southeast Asia [23]. This is further corroborated by findings that comorbidities including diabetes and hypertension, explained up to 50% of the survival disparities between the black and white women diagnosed with breast cancer in the United States [24]. Physicians should therefore be mindful that ethnicity might play a role as a prognostic factor in breast cancer through several mechanisms.

In the current study, we have shown that presence of pre-existing CVD such as myocardial infarction and stroke appeared to be consistently associated with lower administrations of adjuvant therapies. Our observation is in keeping with results from a large population-based study in Canada, which also showed that women with breast cancer who had pre-existing myocardial infarction, congestive heart failure, arrhythmia or cerebrovascular accidents were less likely to receive the recommended treatment for their cancer including chemotherapy (OR: 0.56; 95% CI: 0.48–0.66) and radiotherapy (OR: 0.75; 95% CI: 0.67–0.83) [25].

Our findings that clustering of modifiable cardiovascular risk factors such as hypertension, diabetes, obesity and dyslipidaemia was not associated with administration of most adjuvant therapies corroborate the notion that these risk factors are presently not factored into cancer treatment decision-making [9]. This however is not unexpected given the prevailing uncertainties on how information on conventional cardiovascular comorbidities including hypertension and diabetes may be useful in improving adjuvant treatment decision-making in oncology practices. The lack of association between cardiovascular risk and administration of cancer therapies does not allude to a need to change clinical practices in terms of prescribing adjuvant therapies. Instead, our findings support the pressing need to develop and facilitate stringent cardiac follow-up, surveillance of cardiac-related complications and collaborative multi-disciplinary care. To this end, cardiotoxicity prediction models may offer an avenue to incorporate data on patient-related cardiovascular factors with cancer treatment-related factors leading to estimation of cardiotoxicity scores to facilitate risk stratification of newly diagnosed cancer patients. Such an approach may potentially enable early identification of high-risk individuals whom may benefit from optimisation of anticancer thera pies, early cardiology referral, close cardiovascular follow-up and participation in cardiac rehabilitation programmes (e.g. structured exercise therapy and nutritional counselling) [8].

However, there is presently an unmet need for accurate cardiovascular prognostic prediction rules for cancer patients [10]. For instance, while the staggering burden of cardiovascular risk factors at initial diagnosis coupled with administration of anthracyclines appears to put women with breast cancer in the present study at high *a priori* risks of developing cardiotoxicity, the Cardiotoxicity Risk Score that we had used to estimate the above is yet to undergo external validation [11]. It must also be noted that in order to develop accurate cardiotoxicity risk prediction models for use in oncology practices, high-quality longitudinal data that include detailed data on patients’ cardiovascular profiles are needed apart from their cancer specific data. These will not only allow investigators to verify and quantify the prognostic impact of the individual cardiovascular risk factors on occurrence of cardiotoxicity, and overall and cancer-specific mortalities prior, but also enable the external validation of newly developed cardiotoxicity risk models.

The strengths of the current study include our prospective design where cardiovascular comorbidities and their influence on adjuvant treatment administration have been examined in an unselected cohort of newly diagnosed breast cancer patients, in which the biomarkers were measured at baseline prior to adjuvant treatment decision-making. Nonetheless, while we are in a position to follow-up patients over long term to measure the association between the individual cardiovascular risk factors and survival outcomes, we are unable to measure the incidence of cardiotoxicity *per se*, and other cardiac outcomes in this inception cohort study due to a lack of resources.

## Conclusion

While many professional guidelines including those from the American Society of Clinical Oncology [12] and the European Society of Medical Oncology [26] have recommended routine cardiovascular evaluation of adult patients with cancer prior to initiation of potentially cardiotoxic therapies, the present findings allude to a gap in knowledge on whether data on burden of traditional cardiovascular risk factors may be useful in optimisation of cancer treatment decision-making. Although use of cardiotoxicity risk prediction tools may aid the above, more research is needed in the development and validation of accurate tools.

From a regional perspective, this study highlights the pressing need to accelerate the establishment of coordinated partnerships between the oncology and cardiology specialities to improve cardiovascular outcomes following breast cancer in the low- and middle-income settings. Although it is currently unclear whether aggressive management of modifiable risk factors of CVD may reduce the risk of cancer therapy-induced cardiotoxicity, it is conceivable that optimal control of these factors may lower the risk of cardiovascular events leading to improved overall survival following breast cancer [7, 21–22]. Notably, in resource-limited settings, amidst the shortage of oncologists and cardiologists, primary care physicians might potentially play an essential role in managing risk factors of CVD in cancer survivors.

## Conflicts of interest

The authors have no conflict of interest to report.

## Funding

The authors have no funding to declare for this work.

## Figures and Tables

**Table 1.** Baseline prevalence of cardiovascular disease, and its risk factors in women newly diagnosed with breast cancer.

Cardiovascular profile at initial cancer diagnosis	Overall (*N* = 2,127)		Age at diagnosis with breast cancer								*p* value for *χ*^2^ test
			<40 years (*n* = 230)		40–49 years (*n* = 566)		50–64 years (*n* = 943)		≥65 years (*n* = 388)		
	*n*	Prevalence, % (95% CI)	*n*	Prevalence, % (95% CI)	*n*	Prevalence, % (95% CI)	*n*	Prevalence, % (95% CI)	*n*	Prevalence, % (95% CI)	
Pre-existing CVD^a^	0105	04.9 (4.0–5.9)	003	01.3 (0.0–2.8)	007	01.2 (0.3–2.1)	052	05.5 (4.1–7.0)	043	11.1 (8.0–14.2)	<0.001
Hypertension^b^	1013	47.8 (45.7–49.9)	034	14.8 (10.2–19.4)	151	026.7 (23.1–30.4)	526	56.1 (52.9–59.3)	302	78.2 (74.1–82.4)	<0.001
Diabetes mellitus^c^	0399	18.8 (17.1–20.5)	005	02.2 (00.3–4.1)	043	07.6 (05.4–9.8)	222	23.5 (20.8–26.2)	129	33.4 (28.7–38.1)	<0.001
Obesity	0382	18.0 (16.3–19.6)	039	17.0 (12.1–21.8)	089	15.7 (12.7–18.7)	199	21.1 (18.5–23.7)	055	14.2 (10.7–17.6)	0.007
Dyslipidaemia^d^	1334	65.3 (63.2–67.3)	112	50.2 (43.7–56.8)	315	58.6 (54.4–62.7)	643	70.3 (67.3–73.2)	264	71.7 (67.1–76.3)	<0.001
Smoking	0036	01.7 (1.1–02.2)	005	02.2 (0.3–4.1)	009	01.6 (0.6–2.6)	015	01.6 (0.8–0.4)	007	1.8 (0.5–3.1)	0.93
Family history of early-onset coronary heart disease^e^	0219	11.2 (9.8–12.5)	017	07.7 (4.2–11.2)	053	10.0 (7.4–12.5)	106	12.2 (10.0–14.4)	043	12.6 (9.1–16.2)	0.16
Number of modifiable CVD risk factors^f^											<0.001
0	0386	18.9 (17.2–20.6)	87	39.0 (32.6–45.4)	155	29.0 (25.1–32.8)	119	13.0 (10.9–15.2)	025	06.8 (4.2–9.4)	
1	0695	34.1 (32.0–36.2)	91	40.8 (34.4–47.3)	220	41.1 (37.0–45.3)	295	32.3 (29.3–35.4)	089	24.2 (19.8–28.6)	
2	0536	26.3 (24.4–28.2)	35	15.7 (10.9–20.5)	112	20.9 (17.5–24.4)	257	28.2 (25.3–31.1)	132	35.9 (31.0–40.8)	
≥3	0421	20.7 (18.9–22.4)	10	04.5 (1.8–7.2)	048	0 9.0 (6.6–11.4)	241	26.4 (23.6–29.3)	122	33.2 (28.3–38.0)	

a Includes angina (44), myocardial infarction (31), stroke (30), atrial fibrillation (3), valvular diseases (3), heart failure (2), cardiomyopathy (1) and rheumatic heart disease (1)

b Data were missing in eight patients. The presented data includes 15 patients who self-reported their hypertension

c Data were missing in four patients. The presented data only includes patients with type 2 diabetes

d Data were missing in 83 patients. The presented data includes 17 patients who self-reported their dyslipidaemia

e Defined as coronary heart disease diagnosed before the age of 60 years in a first-degree relative

f Includes hypertension, diabetes mellitus, obesity, dyslipidaemia and smoking. Eighty-nine patients were excluded due to missing data

**Table 2.** Baseline prevalence of CVD, and its risk factors by ethnicity in women newly diagnosed with breast cancer.

Cardiovascular profile at initial cancer diagnosis	Ethnicity						p value for χ² test
	Malay (*n* = 795)		Chinese (*n* = 1,019)		Indian (*n* = 270)		
	*n*	Prevalence (95% CI)	*n*	Prevalence (95% CI)	*n*	Prevalence (95% CI)	
Preexisting CVD^a^	46	05.8 (4.2–7.4)	35	03.4 (02.3–4.6)	22	08.1 (04.9–11.4)	0.002
Hypertension^b^	424	53.3 (49.9–56.8)	409	40.5 (37.4–43.5)	159	58.9 (53.0–64.8)	<0.001
Diabetes mellitus^c^	188	23.7 (20.7–26.6)	96	009.4 (7.7–11.2)	108	40.0 (34.2–45.8)	<0.001
Obesity	228	28.7 (25.5–31.8)	82	008.0 (6.4–9.7)	66	24.4 (19.3–29.6)	<0.001
Dyslipidaemia^d^	517	65.7 (62.4–69.0)	599	63.1 (60.0–66.2)	194	72.9 (67.6–78.3)	0.02
Smoking	7	00.9 (0.2–1.5)	25	002.5 (1.5–3.4)	2	0.7 (0.0–1.8)	0.02
Family history of early-onset coronary heart disease^e^	102	14.1 (11.6–16.7)	71	007.5 (05.8–9.2)	41	16.1 (11.6–20.7)	<0.001
Number of modifiable CVD risk factors^f^							<0.001
0	133	16.9 (14.3–19.5)	217	23.0 (20.3–25.7)	27	10.2 (6.5–13.8)	
1	226	28.7 (25.6–31.9)	382	40.5 (37.4–43.6)	70	26.3 (21.0–31.6)	
2	210	26.7 (23.6–29.8)	243	25.8 (23.0–28.6)	76	28.6 (23.1–34.0)	
≥3	218	27.7 (24.6–30.8)	101	10.7 (8.7–12.7)	93	35.0 (29.2–40.7)	

a Includes angina, myocardial infarction, stroke, atrial fibrillation, valvular diseases, heart failure, cardiomyopathy and rheumatic heart disease

b Data missing in eight patients. The presented data includes 15 patients who self-reported their hypertension

c Data missing in four patients

d Data missing in 82 patients. The presented data includes 17 patients who self-reported their dyslipidaemia

e Defined as coronary heart disease diagnosed before the age of 60 years in a first-degree relative

f Includes hypertension, diabetes mellitus, obesity, dyslipidaemia and smoking. Eighty-eight patients were excluded due to missing data for any of the risk factors

**Table 3.** Association between baseline cardiovascular profile and adjuvant treatment administration in 1,876 women with non-metastatic breast cancer.

Cardiovascular profile at initial cancer diagnosis	Adjuvant therapy administration			
	OR for chemotherapy (95% CI)^a^	OR for radiotherapy (95% CI)^b^	OR for endocrine therapy (95% CI)^c^	OR for trastuzumab (95% CI)^d^
**Pre-existing CVD^e^**				
Yes	0.32 (0.17–0.58)	0.63 (0.37–1.08)	0.47 (0.21–1.09)	-
No	1.00	1.00	1.00	-
**Presence of two or more modifiable risk factors of CVD^f^**				
Yes	0.95 (0.71–1.28)	1.12 (0.86–1.44)	1.60 (1.10–2.50)	0.67 (0.39–1.15)
No	1.00	1.00	1.00	1.00
**Family history of early-onset coronary heart disease^g^**				
Yes	0.89 (0.59–1.36)	1.22 (0.83–1.78)	1.02 (0.55–1.88)	1.31 (0.69–2.82)
No	1.00	1.00	1.00	1.00

a This analysis included 1,752 women of whom 1,105 received adjuvant chemotherapy (124 patients with unknown chemotherapy status were excluded). Results were derived using a multivariable logistic regression model including preexisting CVD, presence of ≥2 risk factors of CVD and family history of early-onset coronary heart disease, adjusted for age (years), ethnicity (Chinese, Malay, Indian, others), type of hospital (public, private, university), tumour size category (pT1, pT2, pT3, pT4), lymph node status (pN0, pN1, pN2, pN3), oestrogen/progesterone receptor status (positive, negative), human epidermal growth factor receptor 2 status (positive, negative), tumour grade (I, II, III), adjuvant radiotherapy administration (no, yes), adjuvant endocrine therapy administration (no, yes) and trastuzumab administration (no, yes)

b This analysis included 1,749 women of whom 1,104 received adjuvant radiotherapy (127 patients with unknown radiotherapy status were excluded). Results were derived using a model similar as in (a) but mutually adjusted for adjuvant chemotherapy status, adjuvant endocrine therapy status and trastuzumab administration

c This analysis included 1,790 women of whom 1,115 received adjuvant endocrine therapy (86 patients with unknown endocrine therapy status were excluded). Results were derived using a model similar as in (a) but mutually adjusted for adjuvant chemotherapy status, adjuvant radiotherapy status and trastuzumab administration

d This analysis included 1,834 women of whom 126 received trastuzumab (42 patients with unknown trastuzumab administration status were excluded). Results were derived using a multivariable logistic regression model including presence of ≥2 risk factors of CVD and family history of early-onset coronary heart disease, adjusted for age, type of hospital, human epidermal growth factor receptor 2 status, adjuvant chemotherapy status, adjuvant radiotherapy administration and adjuvant endocrine therapy status

e Comprises angina, myocardial infarction, stroke, atrial fibrillation, valvular diseases, heart failure, cardiomyopathy and rheumatic heart disease

f Includes hypertension, diabetes mellitus, obesity, dyslipidaemia and smoking

g Defined as coronary heart disease diagnosed before the age of 60 years in a first-degree relative

**Table 4.** Predicted risk of cardiotoxicity following adjuvant therapy in women with non-metastatic breast cancer.

	Predicted risk of cardiotoxicity^a^								OR (95% CI)^b^
	Low		Intermediate		High		Very high		
	*n*	%	*n*	%	*n*	%	*n*	%	
Overall	340	19.9	282	16.5	577	33.7	511	29.9	
**Age group**									
<40	29	15.8	9	4.9	114	62.3	31	16.9	6.61 (4.05–10.79)
40–49	113	24.3	32	6.9	214	46.0	106	22.8	3.97 (2.79–5.66)
50–64	171	22.8	103	13.7	202	26.9	274	36.5	2.40 (01.77–3.26)
≥65	027	08.7	138	44.2	047	15.1	100	32.1	1.00
**Ethnicity**									
Malay	076	11.7	085	13.1	249	38.4	238	36.7	01.29 (0.94–1.76)
Chinese	236	29.1	148	18.2	248	30.6	179	22.1	1.00
Indian	023	10.5	041	18.6	070	31.8	086	39.1	01.43 (0.97–2.11)
Others	005	16.1	008	25.8	010	32.3	008	25.8	00.64 (0.28–1.47)
**TNM cancer stage**									
I	179	42.9	111	26.6	088	21.1	039	09.4	1.00
II	115	15.2	118	15.6	284	37.5	240	31.7	04.82 (03.65–6.36)
III	041	07.8	051	09.7	204	38.6	232	43.9	10.13 (07.29–14.07)

a *A priori* risk of cardiotoxicity was estimated using the Cardiotoxicity Risk Score, which incorporates drug-related and patient-related risk factors [11]

b This analysis included 1,702 women of whom 1,088 were predicted to have high/very high of risks of cardiotoxicity (166 patients with lack of data to compute Cardiotoxicity Risk Score were excluded). Results were derived using logistic regression analysis with high/very high risk of cardiotoxicity as outcome of interest, and age, ethnicity, type of hospital and TNM cancer stage as covariates
